# Deciphering Combinations of PI3K/AKT/mTOR Pathway Drugs Augmenting Anti-Angiogenic Efficacy *In Vivo*

**DOI:** 10.1371/journal.pone.0105280

**Published:** 2014-08-21

**Authors:** Temitope Sasore, Breandán Kennedy

**Affiliations:** UCD School of Biomolecular and Biomedical Science, UCD Conway Institute, University College Dublin, Dublin, Ireland; Queen's University Belfast, United Kingdom

## Abstract

Ocular neovascularization is a common pathology associated with human eye diseases *e.g.* age-related macular degeneration and proliferative diabetic retinopathy. Blindness represents one of the most feared disabilities and remains a major burden to health-care systems. Current approaches to treat ocular neovascularisation include laser photocoagulation, photodynamic therapy and anti-VEGF therapies: Ranibizumab (Lucentis) and Aflibercept (Eylea). However, high clinical costs, frequent intraocular injections, and increased risk of infections are challenges related with these standards of care. Thus, there is a clinical need to develop more effective drugs that overcome these challenges. Here, we focus on an alternative approach by quantifying the in vivo anti-angiogenic efficacy of combinations of phosphatidylinositol-3-kinase (PI3K) pathway inhibitors. The PI3K/AKT/mTOR pathway is a complex signalling pathway involved in crucial cellular functions such as cell proliferation, migration and angiogenesis. RT-PCR confirms the expression of PI3K target genes (*pik3ca, pik3r1, mtor* and *akt1*) in zebrafish trunks from 6 hours post fertilisation (hpf) and in eyes from 2 days post fertilisation (dpf). Using both the zebrafish intersegmental vessel and hyaloid vessel assays to measure the in vivo anti-angiogenic efficacy of PI3K/Akt/mTOR pathway inhibitors, we identified 5 µM combinations of i) NVP-BEZ235 (dual PI3K-mTOR inhibitor) + PI-103 (dual PI3K-mTOR inhibitor); or ii) LY-294002 (pan-PI3K inhibitor) + NVP-BEZ235; or iii) NVP-BEZ235 + rapamycin (mTOR inhibitor); or iv) LY-294002 + rapamycin as the most anti-angiogenic. Treatment of developing larvae from 2–5 dpf with 5 µM NVP-BEZ235 plus PI-103 resulted in an essentially intact ocular morphology and visual behaviour, whereas other combinations severely disrupted the developing retinal morphology and visual function. In human ARPE19 retinal pigment epithelium cells, however, no significant difference in cell number was observed following treatment with the inhibitor combinations. Collectively, these results highlight the potential of combinations of PI3K/AKT/mTOR pathway inhibitors to safely and effectively treat ocular neovascularization.

## Introduction

Angiogenesis, the development of new blood vessels from pre-existing vessels, is a cascade of tightly regulated events necessary for several physiological processes such as organ regeneration, female reproductive cycle and repair of damaged tissues [1]. By contrast, aberrant angiogenesis is apparent in pathologies including chronic inflammation, rheumatoid arthritis, tumour growth and psoriasis [2]. In relation to the eye, development of abnormal retinal or choroidal vasculatures and vascular leakage are common phenomenon in prevalent diseases including proliferative diabetic retinopathy (PDR), wet age-related macular degeneration (AMD) and diabetic macular oedema (DMO).

Wet AMD is a condition characterized by choroidal neovascularization (CNV), damage to the retinal pigment epithelium (RPE) and loss of overlying photoreceptors underneath the macula as a result of angiogenesis and oedema from the choroidal vasculature [3], [4]. In PDR, retinal vessels proliferate and can lead to blood leakage into the vitreous (vitreous haemorrhage), retinal swelling (macula edema), and scar tissue leading to retinal detachment.

PDR and wet AMD are responsible for ∼4.89 million and ∼1.51 million cases, respectively, of visual disability and irreversible blindness worldwide [5]. In addition, these complications represent a substantial health burden to the patients, caregivers, health-care system and the economy. In 2007, Preventing Blindness America reported that the direct medical costs of DR and AMD in the US alone, stands at ∼$490 and $570 million, respectively. Although different in aetiology, the aforementioned diseases share common cardinal features: vessel permeability, angiogenesis, inflammation and neurodegeneration. Elevated vascular endothelial growth factor (VEGF) is one of the pathological drivers [6], [7]. Current anti-VEGF therapies for ocular neovascularization include Ranibizumab (Lucentis), a monoclonal antibody fragment [8], [9], Aflibercept (Eylea), a recombinant decoy protein [10], or off-label Bevacizumab (Avastin), a full-length monoclonal antibody [11].

Despite the therapeutic benefits of anti-VEGF biologicals in some patients, rapid drug clearance impedes long-term visual improvements in others [12], [13]. Thus, patients require frequent in-clinic injections of anti-VEGFs into the vitreous of the eye. This standard of care is associated with a high clinical cost, limited patient independence and an increased risk of infection, endophthalmitis, retinal detachment or cataracts [14], [15]. A complementary strategy to circumvent these disadvantages is to develop small molecule, VEGF-independent inhibitors. These could reduce the frequency of intravitreal injections if delivered topically or as sustained release implants.

The phosphatidylinositol 3-kinase (PI3K)/Akt/mammalian target of rapamycin (mTOR) signalling pathway is a candidate therapeutic target for angiogenesis [16], [17]. The PI3K family are closely related lipid kinases regulating cell metabolism, growth, survival, migration and angiogenesis [18], [19]. PI3K family members are sub-classified based on structural features and substrate specificity. Class 1A enzymes form heterodimeric proteins composed of one of three closely related catalytic subunits: (p110α [PIK3CA], p110β [PIK3CB] or p110δ [PIK3CD); associated with p85/p50/p55 regulatory subunit [20], [21]. Class 1B PI3K comprise of the p110γ catalytic subunit associated with a p85 regulatory subunit [22], [21]. The activation of PI3K via receptor tyrosine kinases (RTKs), RAS or G-protein coupled receptors (GPCRs) initiates the recruitment of PI3K subunits at the plasma membrane, facilitating the phosphorylation of phosphatidylinositol 4,5-biphosphate (PIP2) to generate the second messenger phosphatidylinositol-3,4,5-triphosphate (PIP3). The activity of PIP3 is tightly regulated by phosphatase and tensin homologue deleted on chromosome 10 (PTEN). PIP3 triggers the activation of downstream signalling components including AKT which transduces signals to downstream effectors such as FOXO, BAD, glycogen synthase kinase-3 beta (GSK-3β) and mammalian target of rapamycin complex 1 (mTORC1), modulating a host of cellular processes [23].

Given that PI3K/Akt/mTOR pathway drugs have successfully progressed into clinical use for human cancer, similar drugs may have clinical utility in ocular neovascularization [18], [24], [25], [26]. Indeed, the mTOR inhibitors Palomid 529 and Sirolimus (Rapamycin) have progressed to clinical trial for neovascular AMD and diabetic macular oedema, respectively [27], [28]. Thus, the PI3K signalling pathway is an alternative or adjunct target to treat ocular neovascularization.

Drug combinations can produce additive, synergistic or antagonistic effects when a combination produces an effect equal to (additive), greater than (syngergistic), or less than (antagonistic) the sum of the individual drugs [29], [30]. The primary aim of combination therapy is to enhance efficacy, overcome resistance and reduce adverse effects. Based on the extensive molecular interactions within the PI3K/Akt/mTOR pathway, we hypothesised that concurrently inhibiting multiple nodes within the pathway would enhance the anti-angiogenic efficacy of PI3K pathway inhibitors in vivo [21].

The zebrafish (*Danio rerio*) has emerged as an effective model for evaluating the efficacy of anti-angiogenic agents [31]. The complexity of the zebrafish vasculature is akin to that seen in mammals [32]. Also, high fecundity and rapid development permits efficient, cost-effective compound screening in vivo. The small size of the zebrafish embryos facilitates drug administration and enables survival to 3–4 dpf without a functioning vasculature [31]. Finally, the optical clarity of the zebrafish vasculature coupled with transgenic lines fluorescently labelling the vascular endothelium has trivialised the analysis of intersegmental and hyaloid vasculature development [33], [34], [17].

Here, using zebrafish, we quantify the in vivo anti-angiogenic efficacy and safety of a series of commercially available PI3K/AKT/mTOR pathway inhibitors given alone or in combination. We determined that treatment of developing larvae with specific inhibitor combinations: *i*) significantly inhibited developmental angiogenesis, *ii*) exhibited an anti-angiogenic efficacy ∼2 fold higher than the calculated additive effect and *iii*) did not pronouncedly alter ocular morphology or visual behaviour.

## Methods & Materials

### Ethics Statement

All experiments were performed in accordance with ethical exemption approval granted by the UCD animal research ethics committee.

### Zebrafish husbandry

Adult zebrafish (*Danio rerio*) were raised and maintained at 28°C on a 14 h light/10 h dark cycle according to standard procedures [35]. Embryos were obtained through natural spawning and staged by time and morphological criteria [36]. This study utilized transgenic *Tg(fli1:EGFP)* [33] and wild-type Tubingen (Tu) strains of zebrafish.

### Drug treatment

Embryos were placed 5 per well in 48-well plates containing 400 µl of embryo medium/0.1% DMSO. Test compounds were added directly to the embryo medium. For intersegmental vessel (ISV) angiogenesis assay, embryos were treated from 6–24 hpf and from 2–5 dpf for hyaloid vessel (HV) angiogenesis assay [17]. Larvae were left to incubate with drugs at 28°C on a 14 h light/10 h dark cycle, euthanized and fixed with 4% paraformaldehyde (PFA) in PBS at room temperature for 2 hours before analysis. Drugs were purchased from the following: Rapamycin (Selleckchem S1039), NVP-BEZ235 (Selleckchem S1009), PI-103 (Selleckchem S1038), LY294002 (Sigma L9908), A66 (Selleckchem S2636), WYE125132 (Selleckchem S2661), Palomid 529 (Selleckchem S2238), XL147 (Selleckchem S1118) and XL765 (Selleckchem S1523). 2–5 dpf zebrafish larvae were euthanized in ice-cold PBS, or 4% PFA, or lysis buffer.

### Quantification of intersegmental and hyaloid vessel development

After fixation, larvae were washed three times with PBS. Prior to anti-angiogenic assessment, both control- and treated-larvae were briefly screened for any morphological defects using an Olympus SZX10 microscope. To assess anti-angiogenic activity, embryos were screened for inhibition of intersegmental vessel growth using the following critieria: (a) absence of intersegmental vessel and/or (b) incomplete sprouting of intersegmental vessel from dorsal aorta (DA) to dorsal longitudinal anastomic vessel (DLAV) [37]. For hyaloid vasculature analysis, zebrafish lenses were dissected and mounted on depression microscope slides. The hyaloid vessel plexus surrounding the lens were examined by flourescence microscopy using an Olympus SZX10 microscope. To determine anti-angiogenic activity, treated-larvae were screened for inhibition of hyaloid vessel growth under the following critieria: (a) reduction of primary branches emerging from optic nerve head and/or (b) altered branching pattern. The amount of intersegmental and hyaloid vasculature per treated larvae was quantified by direct observation using an Olympus SZX10 fluorescent microscope.

### Combination Treatment with PI3K/Akt/mTOR inhibitors

In order to uncover drug combinations with additive, synergistic or antagonist properties, we compared the anti-angiogenic effects of the drug combination tested at 5 µM of each drug (actual) versus i) the expected additive effect based on the sum of the effects obtained when testing 5 µM of each drug separately (theoretical) and ii) the effect of 10 µM of either drug tested alone.

### Optokinetic response assay

Visual behaviour was assessed by the optokinetic response (OKR) assay similar to previously described [38]. Treated 5 dpf larvae were immobilized in a petri dish containing Embryo Medium/9% methylcellulose and placed in a black and white stripe drum of 99% contrast and 18° per stripe. The number of eye saccades in response to the rotating drum at 18 rpm, 30 s clockwise and 30 s anti-clockwise was manually counted.

### Histological analysis

Following treatment, adult *Tu* zebrafish larvae were fixed with 4% PFA and 2.5% gluteraldehyde in 0.1 M Sorenson phosphate buffer (pH 7.3) overnight at room temperature. This was followed by one hour post-fixation in 1% osmium tetroxide in 0.1 M Sorenson phosphate buffer at room temperature. Samples were then dehydrated by graded series of ethanol (25%, 50%, 75% & 100%) and embedded in epon resin. Using a diamond knife and a Reichert-Jung Ultracut E microtome, specimens were cut to semi-thin (1 µm) sections, post-stained in toluidine blue and imaged using a Leica DMLB bright field illumination microscope and a Leica DFC 480 camera.

### RNA extraction and cDNA synthesis

Embryos were collected at 7 developmental stages (6, 13, 18, 24, 48, 72 and 120 hpf). Total RNA was extracted from pooled embryos (6, 13, 18 and 24 hpf) and dissected eyes (48, 72 and 120 hpf) using RNeasy Mini Kit (Qiagen, Hilden, Germany) in an RNase-free environment. RNA quality and concentration was determined using a NanoDrop ND-1000 (ThermoScientific). Reverse transcription of total RNA to single-strand cDNA was performed using the RT-PCR SuperScript III First-Strand Synthesis system and random hexamers (Invitrogen) according to manufacturer's instructions. Negative controls were synthesized using the same reaction without SuperScript III enzyme. Subsequent RT-PCR was carried out using the T100 Thermo cycler (Bio-Rad). The RT-PCR products were analysed on 2% agarose gels. Bands were visualized using SYBR safe DNA gel stain (Invitrogen). Expression levels were normalised to the house-keeping gene β-actin. The primer sets (Table 1) or target genes were designed using Primer-BLAST and synthesised by Eurofins MWG Operon (Germany).

**Table 1:** Sequence of primers used in PCR amplification of specific target genes.

Gene		Sequence 5′ – 3′	Product Size (bp)
*PIK3CA*	**F R**	CGCAATGAGAGGATGAGCGA ACGCTGTCACGATGGAACAA	126
*PIK3R1*	**F R**	ACATGGCTCTGCAAGATGCT GGAGGCATCTCGGACCAAAA	110
*mTOR*	**F R**	AGATCATCAACCGAGTGCGG AGGGCACCATCCAATGTAGC	146
*AKT1*	**F R**	TCGGCAGGTGTCTTCTCAAT ACCCATTGCCATACCACGAG	141

### Quantitative Real-time PCR validation

Real-time PCR was carried out as previously described [39] using the cDNA samples described above. Three biological replicates were used for all time points. Real-time PCR was performed using an ABI 7900HT Sequence Detection System. Expression levels were normalized to 18s rRNA. SYBR Green was used as the reporter in all reactions and Taqman probe in the 18s control. Real-time data were normalized according to 18s rRNA and standardized to lowest abundance value.

### Cell culture

Human retinal pigment epithelium (ARPE19) cells were obtained from the American Type Culture collection (ATCC) (Rockville, MD). Cells were cultured in Dulbecco's Modified Eagle's Medium/Nutrient Mixture F-12 Ham (DMEM, Sigma Aldrich) supplemented with 10% fetal bovine serum (FBS), 2 mM L-glutamine, 100 units/ml penicillin, 1 mg/ml streptomycin (Invitrogen) and maintained at 37°C in a humidified 95/5% air/CO_2_ atmosphere. DMEM medium was changed every two days, cells grown to 90–95% confluency and trypsinized with trypsin EDTA (Sigma).

### MTT cell viability assay

ARPE19 cells were seeded at a density of 1.4×10^4^ cells per well of a 96-well plate. The cells were grown to 70% confluency and subsequently serum-starved in minimum media for 24 hours. Cells were treated with PI3K/Akt/mTOR pathway inhibitors individually and in combination for 24 h and 48 h. 0.1% DMSO was used as control. MTT cell viability assay was preformed according to the manufacturer's instructions (Roche).

### Reagent and Antibodies

Primary antibodies used in Western blot analysis include rabbit polyclonal phospho-Akt (#9275), Akt (#9272), phoshpo-p70S6k (#9205), p70S6k (#2708) which were purchased from Cell Signalling Technologies. Anti-rabbit horseradish peroxidase (HRP)-labelled secondary antibody (NA934) was purchased from GE Healthcare.

### Preparation of tissue sample for protein extraction and immunoblot analysis

Zebrafish larvae treated from 2 to 5 dpf were first washed with embryo medium and then immobilized in ice-cold PBS. To increase signal from specific cellular protein, the larvae were de-yolked in ice-cold PBS and incubated in ice-cold lysis buffer. Protein was harvested in lysis buffer consisting of Tris/HCl, pH 7.5 (50 mM), 0.25% sodium deoxycholate, 150 mM NaCl, 1 mM EGTA, 1 mM Na_3_VO_4_, 1 mM NaF, 1% (v/v), 1 mM PMSF supplemented with protease inhibitor cocktail (Sigma P2714) and phosphatase inhibitor cocktail (Sigma P5726). Following sonication, nuclear and cellular debris were removed by centrifugation at 12000 g for 15 min at 4°C. Protein concentration was quantified by Bradford assay (Bio-Rad Laboratories). Samples were resolved by SDS/PAGE, transferred to a polyvinyl difluoride (PVDF) membrane and blocked for 1 hour in PBS-T (Phosphate-buffered saline containing 0.1% Tween 20) and 5% (w/v) skimmed milk. Membranes were incubated overnight with primary antibody at 4°C before incubation with HRP-conjugated secondary antibody incubations at room temperature for 1 hour. Chemiluminescent detection using Amersham ECL Prime detection reagent (GE Healthcare RPN2232) allowed for visualisation of the protein bands. Quantitative measurement of phosphorylated protein level was performed using ImageJ software (National Institutes of Health). Results are presented as relative densitometry ratios of phospho-protein relative to band intensity of total protein.

### Statistical analysis

Bar graphs were generated using Microsoft Excel 2010 to illustrate activity in each treatment group. Results are presented as mean ± s.e.m. Significance of difference between mean values was evaluated by analysis of variance (one-way ANOVA) with subsequent comparison by Dunnet's *post hoc* test. Student's t-test was used to compare groups.

## Results

### PI3K/Akt/mTOR genes are expressed in developing zebrafish embryos and eyes

**Figure 1:**
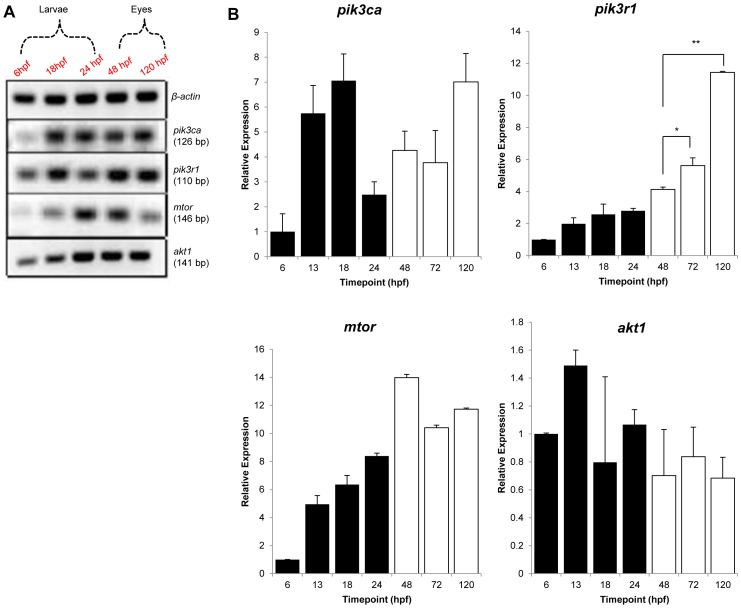
PI3K/Akt/mTOR gene expression in developing Tg(fli1:EGFP) zebrafish. (A) RT-PCR and (B) qPCR examined the mRNA levels of zebrafish *pik3ca, pik3r1, mTOR and akt1* genes. Embryos were harvested at 6, 13, 18 or 24 hours post fertilization (hpf) and eyes at 48, 72 or 120 hours post fertilization (hpf). (A). Relative expression, normalised to *18S rRNA*, of *pik3ca, pik3r1, mTOR and akt1* mRNA levels in developing zebrafish embryos (black columns) and eyes (white columns) expressed relative to the 6 hpf stage. Results are expressed as mean ± S.D. (*n* = 3), *P<0.05, **P<0.01.

As the test drugs are PI3K/Akt/mTOR pathway inhibitors, we first determined whether zebrafish *pik3ca, pik3r1, mTOR and akt1* genes are expressed in developing whole zebrafish embryos and isolated eyes. Fig 1A shows amplification of the expected RT-PCR products (126, 110, 146 and 141 bp, respectively) confirming these genes are expressed in 6, 18 and 24 hpf zebrafish embryos and 48 and 120 hpf zebrafish eyes. These results were corroborated by quantitative RT-PCR; which in general indicated higher gene expression levels as development progressed and that significantly higher levels of *pik3r1* were expressed in 5 dpf eyes versus earlier time-points (Fig 1B).

### Combinations of PI3K/Akt/mTOR pathway inhibitors are potent inhibitors of intersegmental vessel (ISV) angiogenesis

**Figure 2:**
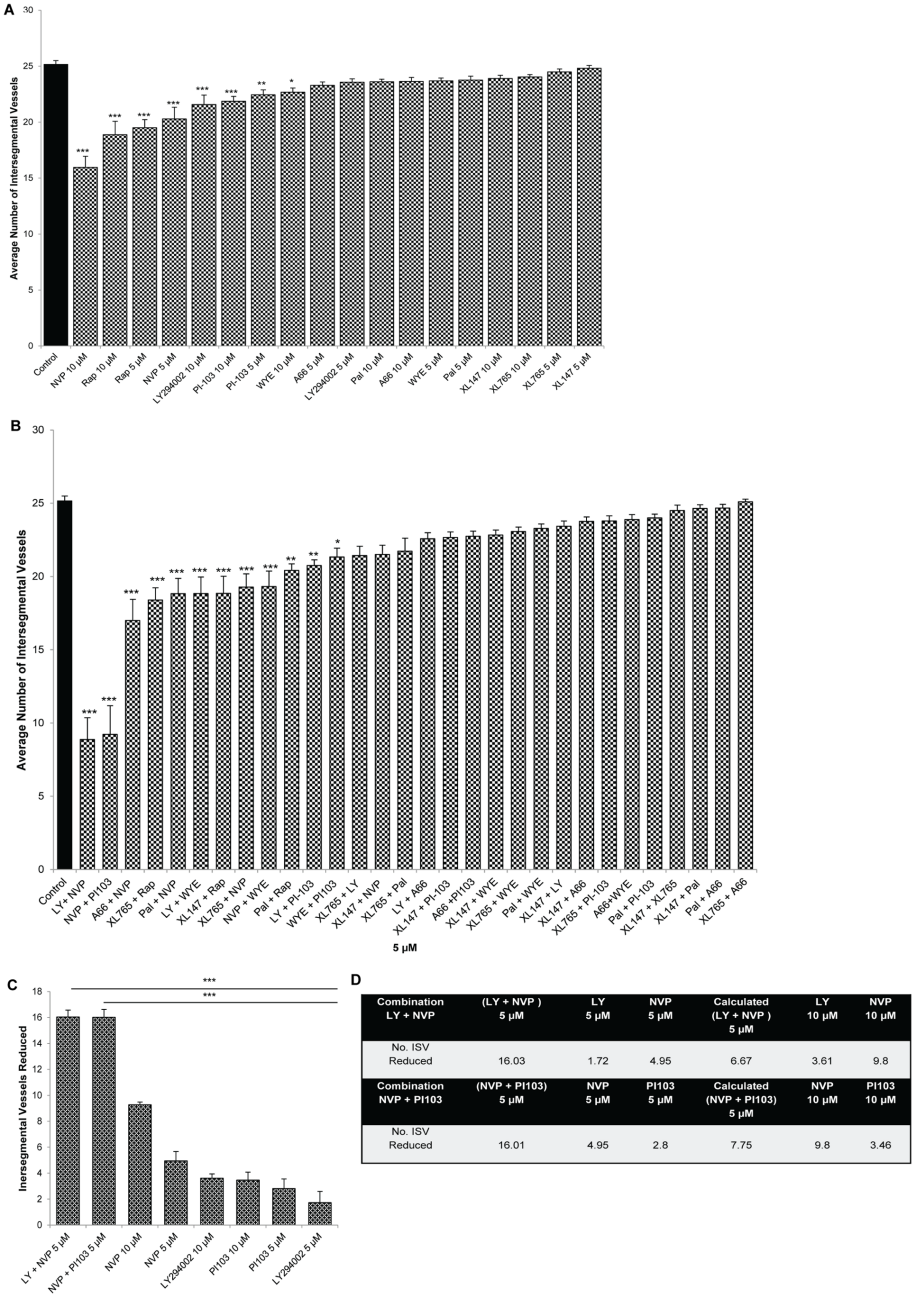
PI3K/Akt/mTOR inhibitors inhibit developmental angiogenesis of the intersegmental vasculature. Multiple PI3K inhibitors were screened for effects on developmental angiogenesis of the trunk/tail vasculature in vivo. 6 hours post fertilisation (hpf) Tg(*fli1:EGFP*) embryos were treated with the specified compounds and the number of ISV quantified at 2 dpf. Bar graphs show ranking efficacy of individual PI3K/Akt/mTOR inhibitors at 5 µM and 10 µM (A), combinations at 5 µM (B), and top two PI3K/Akt/mTOR combinations compared to individual drugs alone (C). Table summarizing effects of inhibitors at 5 µM or 10 µM, actual and calculated combination on ISV angiogenesis (D). Data are means ± s.e.m. (*n* = 24–30); *P<0.05, **P<0.01, ***P<0.001. * symbolize significant differences from LY294002 + NVP-BEZ235-treated larvae or NVP-BEZ235 + PI-103-treated larvae.

**Figure 3:**
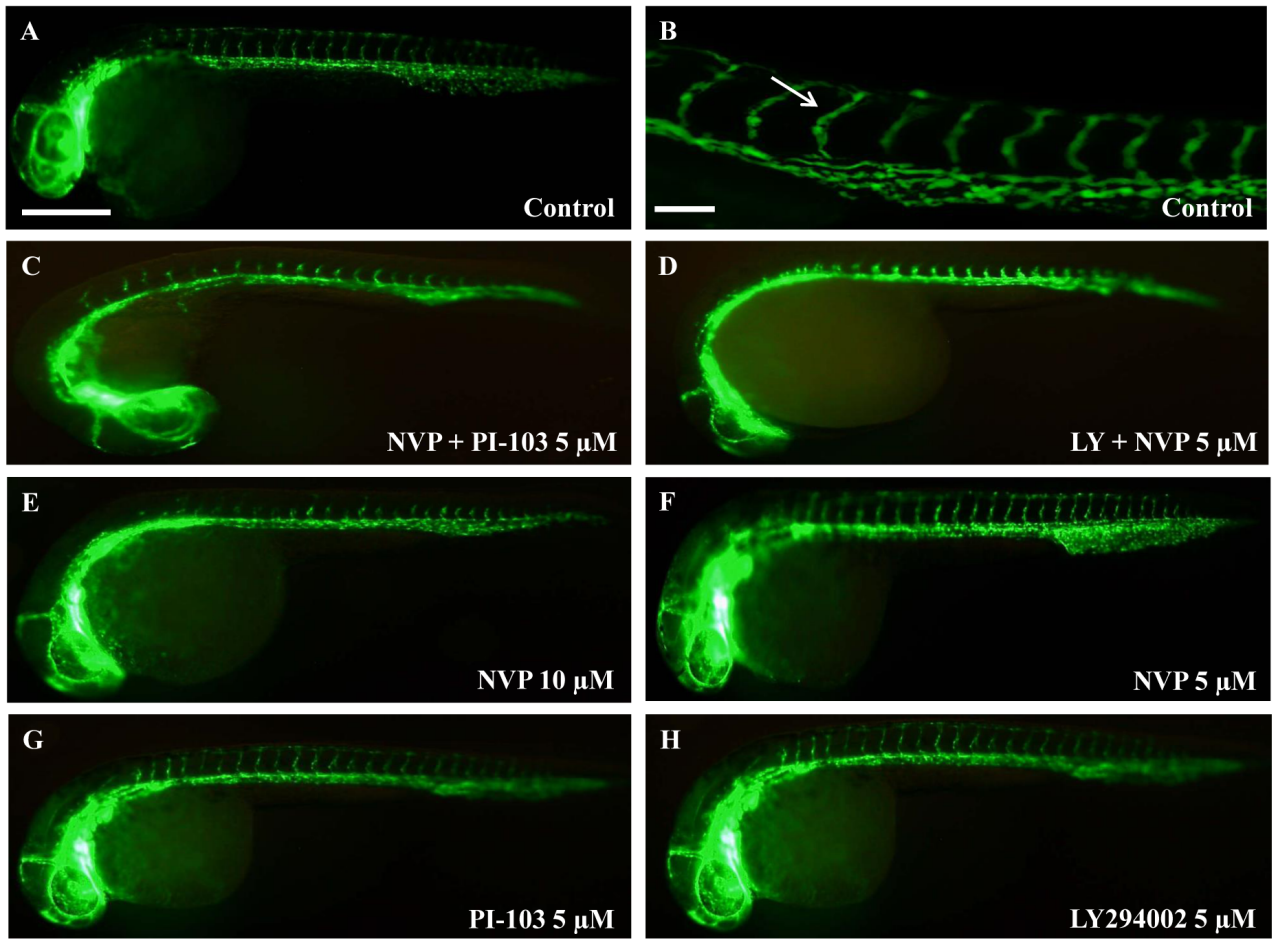
PI3K/Akt/mTOR inhibitors inhibit developmental angiogenesis of the intersegmental vasculature. Representative images demonstrating anti-angiogenic effects of PI3K/Akt/mTOR inhibitors on ISV development. In 0.1% DMSO-treated control larvae ∼25 ISVs pairs develop (A) and high magnification (B). 5 µM NVP-BEZ235 + PI-103 (C), 5 µM LY294002 + NVP-BEZ235 (**D**) and 10 µM NVP-BEZ235 (**E**) exhibited significant anti-angiogenic effects compared to individual drugs at 5 µM (F–H). White arrows indicate the ISVs sprouting from the dorsal aorta (DA) to the dorsal longitudinal anastomic vessel (DLAV). Scale bar in A is 500 µm and in B is 100 µm (**B**). n = 25–30.

Using *Tg(fli1:GFP)* embryos, a panel of nine PI3K/Akt/mTOR pathway inhibitors were screened for anti-angiogenic activity using the ISV assay. Drugs were first tested at 5 or 10 µM individually and this primary screen shows that 5 or 10 µM NVP-BEZ235, 5 or 10 µM Rapamycin, 5 or 10 µM PI-103, 10 µM LY294002 or 10 µM WYE125132 exhibit a modest, but significant, inhibition in ISV developmental angiogenesis of (Fig 2A, Fig. 3E–H).

We then assessed the anti-angiogenic efficacy of drug combinations at 5 µM each (Fig. 2B–3). Amongst the thirty combinations screened, 5 µM LY294002 + NVP-BEZ235 or 5 µM NVP-BEZ235 + PI-103 most potently inhibit ISV angiogenesis; up to ∼70% of DMSO-treated controls (Fig 2B, Fig. 3). Several other combinations also exhibit significant, but less pronounced inhibition (Fig. 2B, Fig. 3). The combination of 5 µM LY294002 + NVP-BEZ235 is significantly more anti-angiogenic than either 5–10 µM LY294002 or 5–10 µM NVP-BEZ235 tested alone (Fig 2C). Similarly, 5 µM NVP-BEZ235 + PI-103 is significantly more anti-angiogenic than either 5–10 µM NVP-BEZ235 or 5–10 µM PI-103 alone (Fig 2C). To investigate if these drug combinations were exerting additive, or greater than additive effects, we compared the anti-angiogenic activity of the combinations tested at 5 µM of each drug (actual) versus the expected additive effect calculated from the sum of each drug tested at 5 µM alone. 5 µM LY294002 + NVP-BEZ235 or 5 µM NVP-BEZ235 + PI-103 produce an anti-angiogenic effect significantly greater than their calculated additive effect (Fig 2C–2D, Fig. S1A). Notably, these 5 µM combinations are also significantly more effective than testing either drug alone at 10 µM (Fig. 3).

To confirm that the active drugs modulated the PI3K/Akt/mTOR pathway, the expression levels of phosphorylated p70S6 kinase (p-p70S6k) and Akt (p-Akt) were assessed in whole treated zebrafish embryos by Western blot. At the Akt node, the individual drugs but not the combinations modestly reduce p-Akt levels (Fig S2). However, further downstream in the pathway, a significant decrease of greater than 60% of p-p70S6k levels was observed with most individual and combination drugs.

### Combinations of PI3K/Akt/mTOR pathway inhibitors are potent inhibitors of hyaloid vessel (HV) angiogenesis

**Figure 4:**
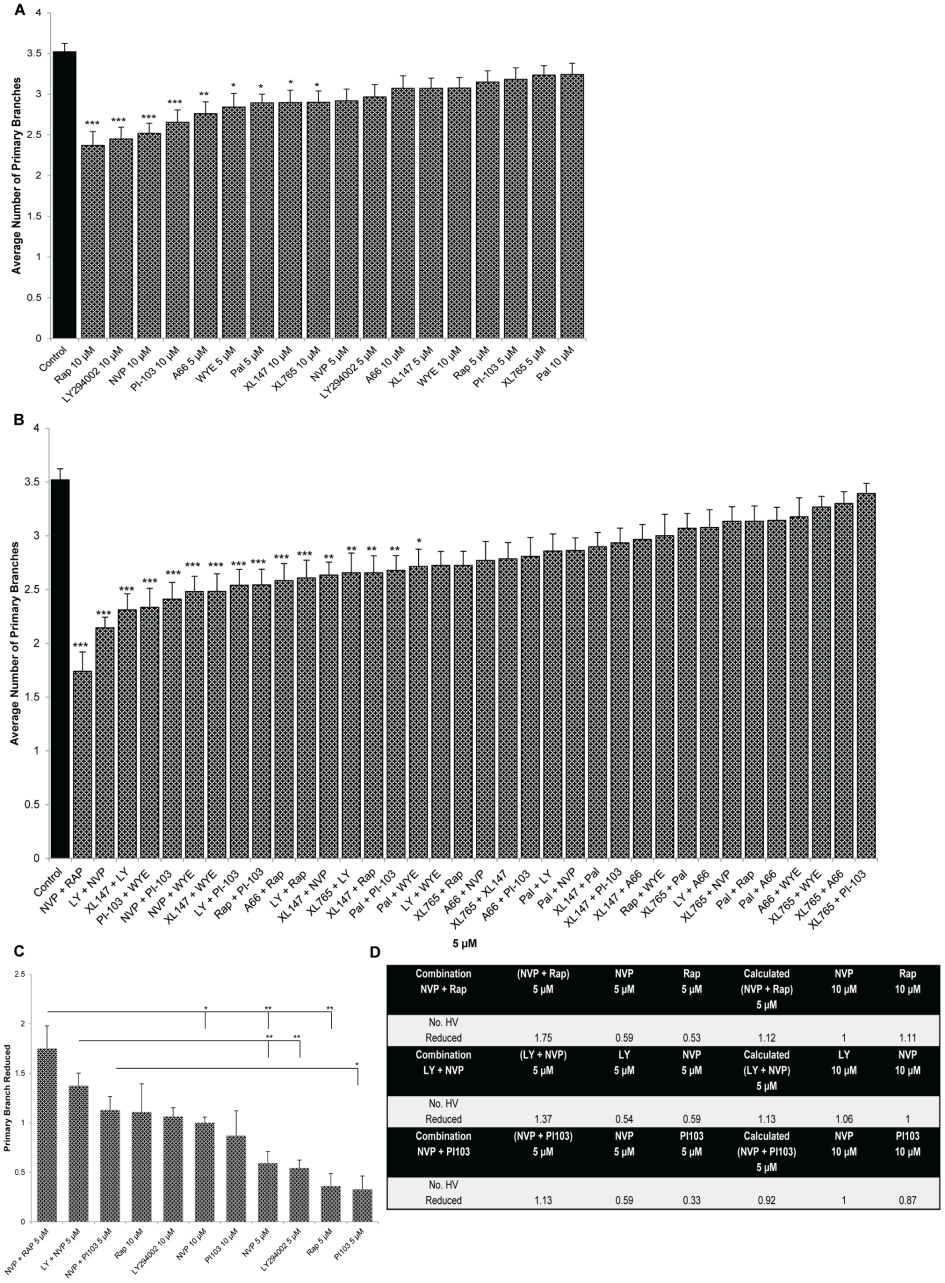
PI3K/Akt/mTOR combinations inhibit developmental angiogenesis in the eye. Tg(*fli1:EGFP*) larvae were treated with multiple PI3K/Akt/mTOR inhibitors from 2 dpf and the number of primary HV branches quantified at 5 dpf. Bar graphs show ranking efficacy of individual PI3K/Akt/mTOR inhibitors tested singly at 5 µM or 10 µM (**A**), tested at 5 µM combinations (**B**) and the top three PI3K/Akt/mTOR combinations compared to individual drugs alone (**C**). Table summarizing effects of treatments at 5 µM or 10 µM, and the actual or calculated combination reductions in HV angiogenesis (**D**). Data are means ± s.e.m. (*N* = 26–30). *P<0.05, **P<0.01 & ***P<0.001. * symbolize significant differences from NVP-BEZ235 + Rapamycin, LY294002 + NVP-BEZ235-treated larvae or NVP-BEZ235 + PI-103-treated larvae.

**Figure 5:**
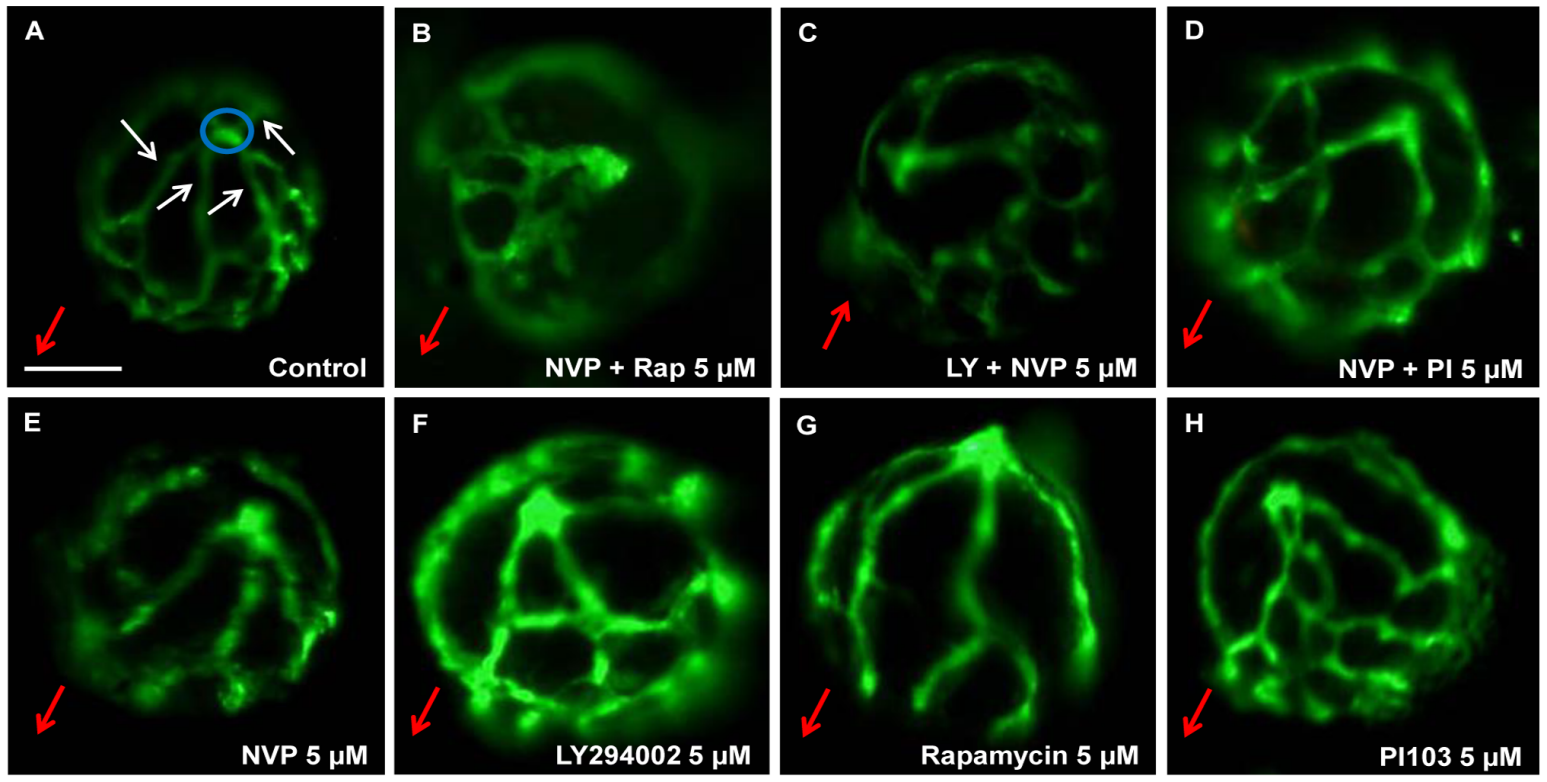
Qualitative effects of PI3K/Akt/mTOR inhibitors on ocular angiogenesis. Representative images demonstrating anti-angiogenic effects of PI3K/Akt/mTOR inhibitors on development of HV. 5 µM NVP-BEZ235 + Rapamycin (B), 5 µM LY294002 + NVP-BEZ235 (C) and 5 µM NVP-BEZ235 + PI103 (D) exhibited significant reductions in primary HV branch number compared to 0.1% DMSO-treated larvae (A). Small, but significant differences were observed in the number of primary branches and overall hyaloid vasculature patterning in 5 µM NVP-BEZ235, 5 µM LY294002, 5 µM Rapamycin and 5 µM -PI103-treated larvae (E-H) compared to control (A). Blue circles depict the optic nerve head. White arrows label primary HV branches emanating from the optic disc at the back of the lens. Red arrows indicate the lens orientation pointing in the direction from the optic disk to the lens. Scale bar 100 µm. *N* = 26–30.

To complement the ISV screen, the nine PI3K/Akt/mTOR pathway inhibitors were screened for anti-angiogenic activity in the zebrafish eye by assessing HV development (Fig. 4–5). 10 µM Rapamycin, 10 µM LY294002, 10 µM NVP-BEZ235, 10 µM PI-103, 5 µM WYE-125132, 5 µM Pal529, 10 µM XL147, 10 µM XL765 and 5 µM NVP-BEZ235 modestly, but significantly, inhibit developmental angiogenesis of the primary HV branches (Fig 4A, Fig 5E–H).

As above, the anti-angiogenic efficacy of PI3K/Akt/mTOR drug combinations at 5 µM was then determined (Fig. 4B, Fig. 5B–C). 5 µM NVP-BEZ235 + Rapamycin, or 5 µM LY294002 + NVP-BEZ235 most strongly inhibit HV development; up to ∼40–60% of control (Fig. 4B). Two combinations, 5 µM NVP-BEZ235 + Rapamycin, and 5 µM LY294002 + NVP-BEZ235 are significantly more anti-angiogenic than 5 µM of either drug alone (Fig. 4C). The combinations also elicit a response greater than the calculated additive effect or to 10 µM of either drug (Fig. 4C–4D, Fig. S1B).

### PI3K/Akt/mTOR Drug Combinations and Ocular Safety

**Figure 6:**
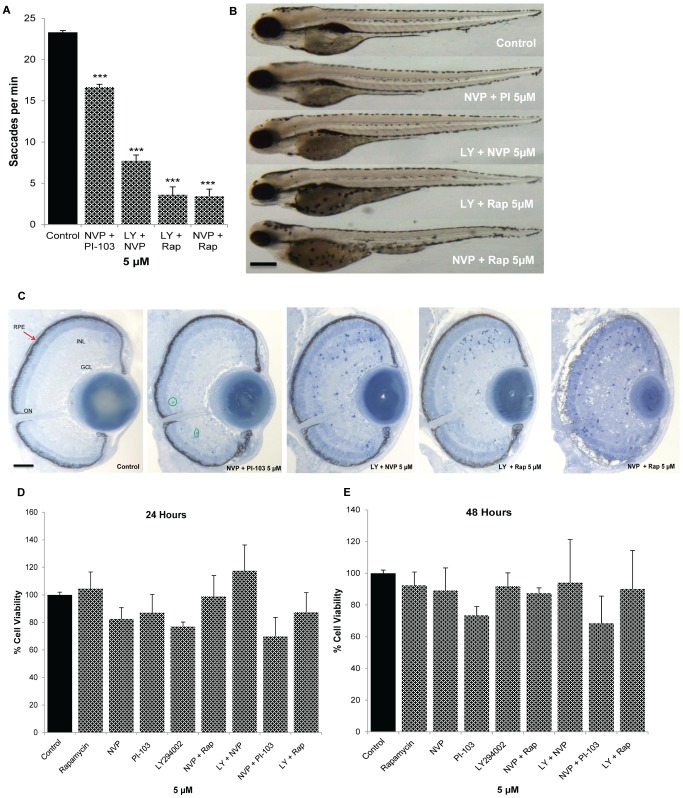
Safety pharmacology of PI3K/Akt/mTOR drug combinations in the zebrafish eye and human ARPE19 cells. 2 dpf larvae were treated with the most potent PI3K/Akt/mTOR inhibitors or control (0.1% DMSO) and eye saccades per minute quantified at 5 dpf (**A**). All PI3K/Akt/mTOR inhibitors reduce the number of saccades per minute compared to control but 5 µM NVP-BEZ235 + PI-103 larvae have a largely intact OKR. Representative images of the gross morphology of treated larvae (**B**). The most potent drug combinations exhibited little to no effect on overall morphology except for 5 µM NVP-BEZ235 + PI-103 which resulted in reduced eye size. Representative light microscopy images of treated larval eyes (**C**). Histological analysis was conducted on 1 µm sections of PI3K/Akt/mTOR-treated larvae at 5 dpf with ten sections analyzed per treatment. Retinal morphology appeared as normal in 5 µM NVP-BEZ235 + PI-103 treated larvae, except for a small number of dying cells. Increased number of apoptotic cells was observed in 5 µM LY294002 + NVP-BEZ235 or 5 µM LY294002 + Rapamycin treated larvae. In particular, 5 µM NVP-BEZ235 + Rapamycin (5 µM) exert adverse effects on retinal lamination, RPE, lens and optic nerve integrity. Effects of PI3K/Akt/mTOR inhibitors on viability of human RPE cells (ARPE19) (**D**–**E**). Confluent cells were treated with either 0.1% DMSO or PI3K/Akt/mTOR inhibitors individually or in combination (5 µM) for 24 h (**D**) and 48 h (**E**) and cell viability was measured by MTT assays. No significant difference was observed in ARPE19-treated cells compared to control. Results are expressed as percentage of control. PI3K/Akt/mTOR inhibitors showed no cytotoxicity on ARPE19 cells. GCL: ganglion cell layer, ONL: outer nuclear layer, INL: inner nuclear layer, ON: optic nerve, RPE: retinal pigment epithelium. Red arrows point to the RPE. Scale bars: **B** = 50 µm, **C** = 100 µm. Data are means ± s.e.m. (*n = 3*). *N* = 20–25 (**A**), *N* = 20 (**B**).

Initial ocular safety pharmacology studies assessed adverse effects on ocular morphology and visual function in zebrafish, and the viability of human RPE cell cultures (Fig. 6). To assess visual function, zebrafish larvae were treated from 2–5 dpf with the most active PI3K pathway drug combinations and the optokinetic response assay conducted (Fig. 6A). 5 µM NVP-BEZ235 + PI-103 resulted in an ∼20% reduction in saccade number whereas the other combinations severely compromised visual function. None of the combinations produce gross changes in larval morphology, with the exception of reduced eye size in larvae treated with 5 µM NVP-BEZ235 + Rapamycin (Fig. 6B). Subsequently, ocular morphology was examined by light microscopy assessing the presence of an optic nerve, RPE integrity, lens size, retinal lamination and the presence of apoptotic cells (Fig. 6C). 5 µM NVP-BEZ235 + PI-103 treated larvae had normal eye histology except for a few apoptotic cells. In contrast, 5 µM LY294002 + NVP-BEZ235 or 5 µM LY294002 + Rapamycin resulted in a marked increase in apoptotic cells and 5 µM NVP-BEZ235 + Rapamycin exhibit severe adverse morphology featuring disrupted retinal cell lamination, pale RPE, small lens, and extensive apoptotic cells. Finally, the anti-angiogenic PI3K pathway drug combinations were tested for adverse effects on the viability of cultured human ARPE19 cells (Fig. 6D–6E). Notably, none of the drug combinations reduced ARPE19 cell number up to 48 hours post-treatment.

## Discussion

The physiological features of zebrafish, particularly its intricate vascular network, miniature size and high fecundity, can accelerate the discovery of enhanced anti-angiogenic agents. Our strategy utilized transgenic Tg*(fli1:EGFP)* zebrafish to screen PI3K/AKT/mTOR pathway inhibitors alone and in combination.

Encouraging pre-clinical and clinical progress has been achieved using drug combinations to treat cancer [40], [41], [42]. Of relevance to PI3K/AKT/mTOR, recent reports showcase anti-tumour activity in vitro and in vivo upon combining rapamycin (mTOR inhibitor inhibitor) with PI-103 (dual PI3K/AKT inhibitor) [43]. As LY294002 (pan-PI3K inhibitor) is anti-angiogenic in the eye, we hypothesized that combinations of PI3K/AKT/mTOR pathway drugs could enhance this ocular response [17]. In agreement, we show here that combinations of i) NVP-BEZ235 + PI-103 (dual PI3K/mTOR inhibitors) or ii) LY294002 + NVP-BEZ235 or iii) NVP-BEZ235 + rapamycin potently inhibit developmental angiogenesis. These combinations exert stronger anti-angiogenic responses compared to equivalent or 2 fold the concentrations of the drugs alone or the calculated additive effects of the drugs.

**Figure 7:**
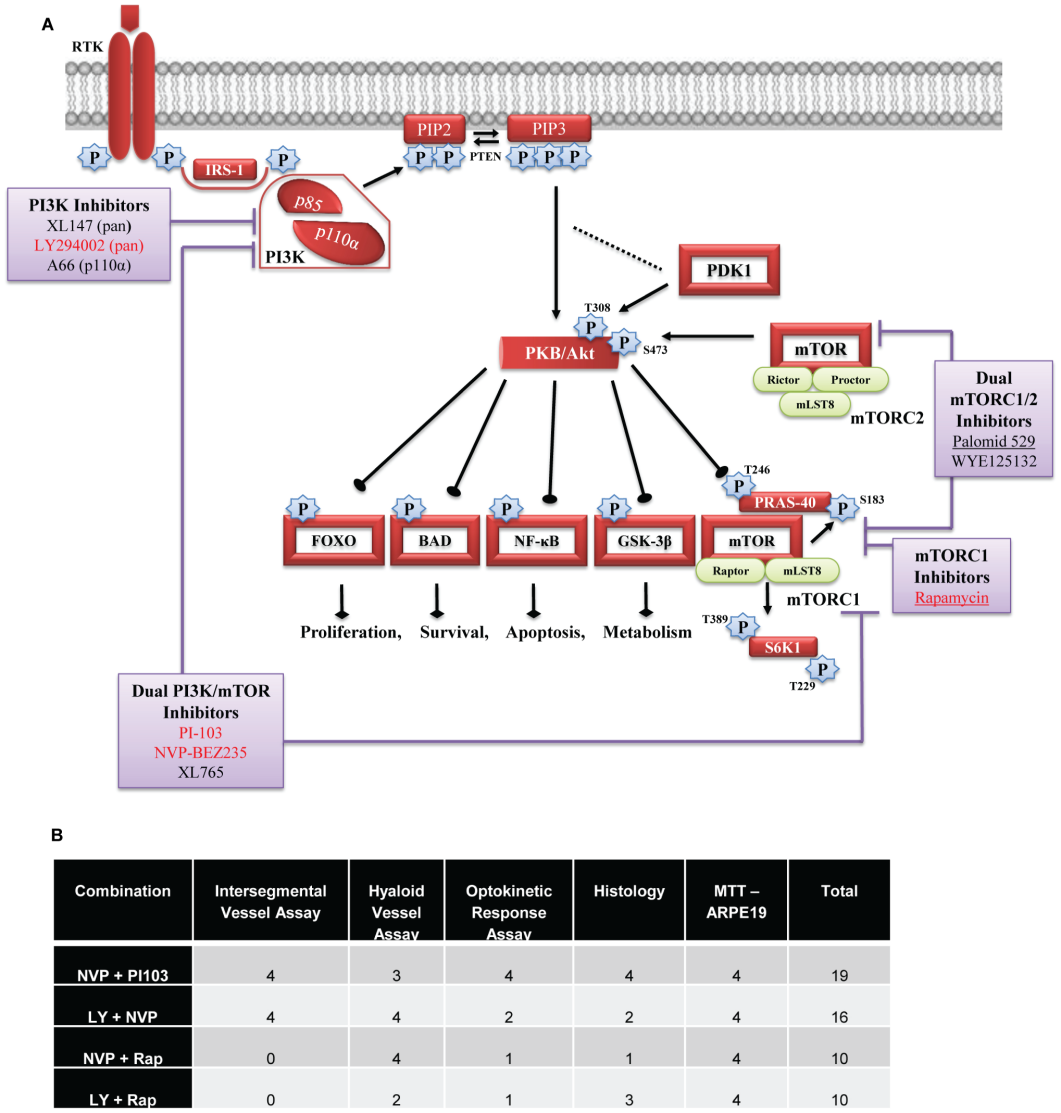
Diagrammatic representation of PI3K/AKT/mTOR signalling pathway detailing tested drug targets and overall ranking table. Upon activation by receptor tyrosine kinase, PI3K lipid kinases are recruited to the plasma membrane where they phosphorylate PIP_2_ to form PIP_3_. Once phosphorylated, the second messenger PIP_3_ can bind and activate AKT which can then trigger an array of downstream signalling events. The site of action tested drugs is indicated and the most effective drugs are depicted in red, and underlined drugs are in clinical trial for ocular indications (A). Table illustrates the overall ranking of the top PI3K/AKT/mTOR inhibitor combinations in the ISV, HV, OKR, LM and MTT experimental assays (B).

The combinations of PI3K pathway inhibitors exerting additive or greater responses is intriguing. Initially, we expected p110α inhibitors would be potent as this subunit is required for angiogenesis [44]. However, the p110α inhibitor A66, did not demonstrate significant anti-angiogenic activity in zebrafish, and tended to be antagonistic in combination. Secondly, we expected “vertical inhibition”, whereby multiple nodes in the pathway are targeted, would produce greater response. This is potentially the case for NVP-BEZ235 + rapamycin which exclusively inhibit p110_α,β,γ,δ_ or mTOR components, respectively (Fig.7, Fig. S3). In contrast, the other combinations with enhanced anti-angiogenic activity have largely overlapping PI3K/Akt/mTOR targets and IC_50_ values (Fig.7, Fig. S3). The enhanced response achieved by these combinations more likely reflects “horizontal inhibition” wherein other angiogenic pathways are also targeted. NVP-BEZ235, PI-103 and LY2940002 are known (with nanomolar IC_50_ values) to inhibit DNA-activated protein kinase (a PI3K-related kinase family member) [45]. NVP-BEZ235, but not PI-103, inhibits ATM (ataxia telangiectasia, mutated) and ATR (ATM and Rad3-related) protein kinases [46]. PI-103, but not NVP-BEZ235, inhibits mTORC2 [47]. However, this interpretation requires further investigation to tease out relevant cross-talk. Importantly, there is precedence for a similar synergistic effect of PI3K pathway inhibitors in mammalian cancer cells [47]. Combination treatment of PI-103 + rapamycin led to synergistic suppression of melanoma cell viability in vitro. In a human melanoma xenograft model, PI-103 or rapamycin alone produced a modest response, whereas the PI-103 + rapamycin combination significantly reduced tumour growth [47].

Although the efficacy of PI3K/Akt/mTOR inhibitors has been well documented in the treatment of human cancer, their advancement in cancer treatment has been limited due to toxicity which manifests following systemic delivery [48], [49]. However in eye diseases, these drugs may be more suitable as they have potential to be delivered locally via eye drops or intraocular injection, therefore overcoming adverse systemic effects. Further translational development of these anti-angiogenic treatment combinations requires demonstration of safety and efficacy in mammalian eyes. Notably, a number of PI3K/AKT/mTOR pathway inhibitors have progressed through clinical trial with adequate safety profiles [28]. Here, we investigated the safety profile of our most effective combinations in zebrafish. 5 µM NVP-BEZ235 + PI-103 resulted in a mild reduction in visual behaviour and ocular histology, whereas the other combinations produced severe adverse effects. This contributes to our overall ranking of NVP-BEZ235 + PI-103 as the most effective and safest combination (Figure 7B). However, there are significant differences between the developmental assays in zebrafish and the proposed use in adult patient eyes that should not preclude further evaluation of the other combinations. In the zebrafish HV assay, nascent vessels are forming between the lens and retina [34], [17]. Previous studies report that the intraocular vasculature regulates normal retinal development [50], [51]. Therefore, the adverse effects on visual behaviour and retinal histology may reflect a loss of vascular supply or cell-cell interactions. This is distinct from the AMD and PDR patients in whom the goal is to stabilise existing vessels and prevent development of new vessels. In agreement, both rapamycin and LY294002 reduce ocular neovascularisation in mouse models without adverse effects on retinal histology [52], [53]. Furthermore, here we demonstrate that the highest ranking drug combinations do not have cytotoxic effects on human RPE cells in vitro cells that are important regulators of retinal angiogenesis (Figure 6D–6E) [54], [55], [35].

In conclusion, our findings reveal combinations of PI3K/AKT/mTOR pathway inhibitors that additively or synergistically disrupt developmental angiogenesis. Future studies will quantify the effects of the PI3K pathway combinations on human retinal endothelial cells in vitro (*e.g.* viability, migration, proliferation and tubule formation) followed by a re-iteration of the ranking order and evaluation of additive/synergistic anti-angiogenic effects in rodent models of retinal or choroidal neovascularisation.

## Supporting Information

Figure S1 **Combination of PI3K/Akt/mTOR inhibitors exhibit additive or greater than additive anti-angiogenic activity in vivo.** Multiple PI3K pathway inhibitors were screened for effects on developmental angiogenesis in vivo. Tg(*fli1:EGFP*) embryos were treated with drugs alone or in combination from 6–48 hpf (ISV) or from 2–5 dpf (HV). Bar graphs show the ranking efficacy of “experimental observed” versus “theoretically calculated” responses in the ISV (**A**) and HV (**B**) assay. Experimental data (black bar) represent response from 5 µM of an inhibitor combination tested together, while theoretical data (white bar) represents the sum of the responses of the corresponding drug pair tested singly. Those combinations exhibiting anti-angiogenic responses significantly greater than the calculated additive responses are marked by asterisks. Four combinations, 5 µM A66 + Rapamycin, 5 µM A66 + WYE-125132, 5 µM Palomid 529 + PI103 or 5 µM Palomid 529 + A66 exhibit significantly reduced ISV responses compared to their calculated additive response, indicating drug antagonism. Three combinations, 5 µM LY294002 + A66, 5 µM Palomid 529 + A66 or 5 µM A66 + WYE-125132 appear to have antagonist effects in the HV assay. Data are means ± s.e.m (*n* = 24–30). *P<0.05, **P<0.01 & ***P<0.001.
(TIF)

Figure S2 **PI3K/Akt/mTOR inhibitors attenuate p70S6k and Akt activity in zebrafish larvae.** Representative Western blot (**A–B**) and densitometric analysis (**C–D**) of extracts from whole larvae treated from 2–5 dpf with 5 µM NVP-BEZ235, 5 µM PI-103, 5 µM LY294002, 5 µM Rapamycin, 2.5 µM NVP-BEZ235 + PI-103, 2.5 µM LY294002 + NVP-BEZ235 and 2.5 µM NVP-BEZ235 + Rapamycin or DMSO. The vast majority of individual and combination drugs significantly reduced the levels of the downstream target p-p70S6k (Thr389) with only modest or no reductions in the levels of p-Akt (Thr308) which is further upstream. Bar graphs show mean band density normalized relative to p-p70S6k/p70S6k ratio or p-Akt/Akt ratio. Data are means ± s.e.m of 4 independent experiments (*n* = 60–80). *P<0.05 & **P<0.01 relative to vehicle control.
(TIF)

Figure S3 **Chemical structures, molecular weight and IC_50_ values of screened PI3K/AKT/mTOR pathway inhibitors.**
(TIF)
