# Supplementary figures and images for: Deciphering Combinations of PI3K/AKT/mTOR Pathway Drugs Augmenting Anti-Angiogenic Efficacy In Vivo
Source: PLoS One. 2014 Aug 21;9(8):e105280. doi: 10.1371/journal.pone.0105280 (PMC4140730; doi:10.1371/journal.pone.0105280)

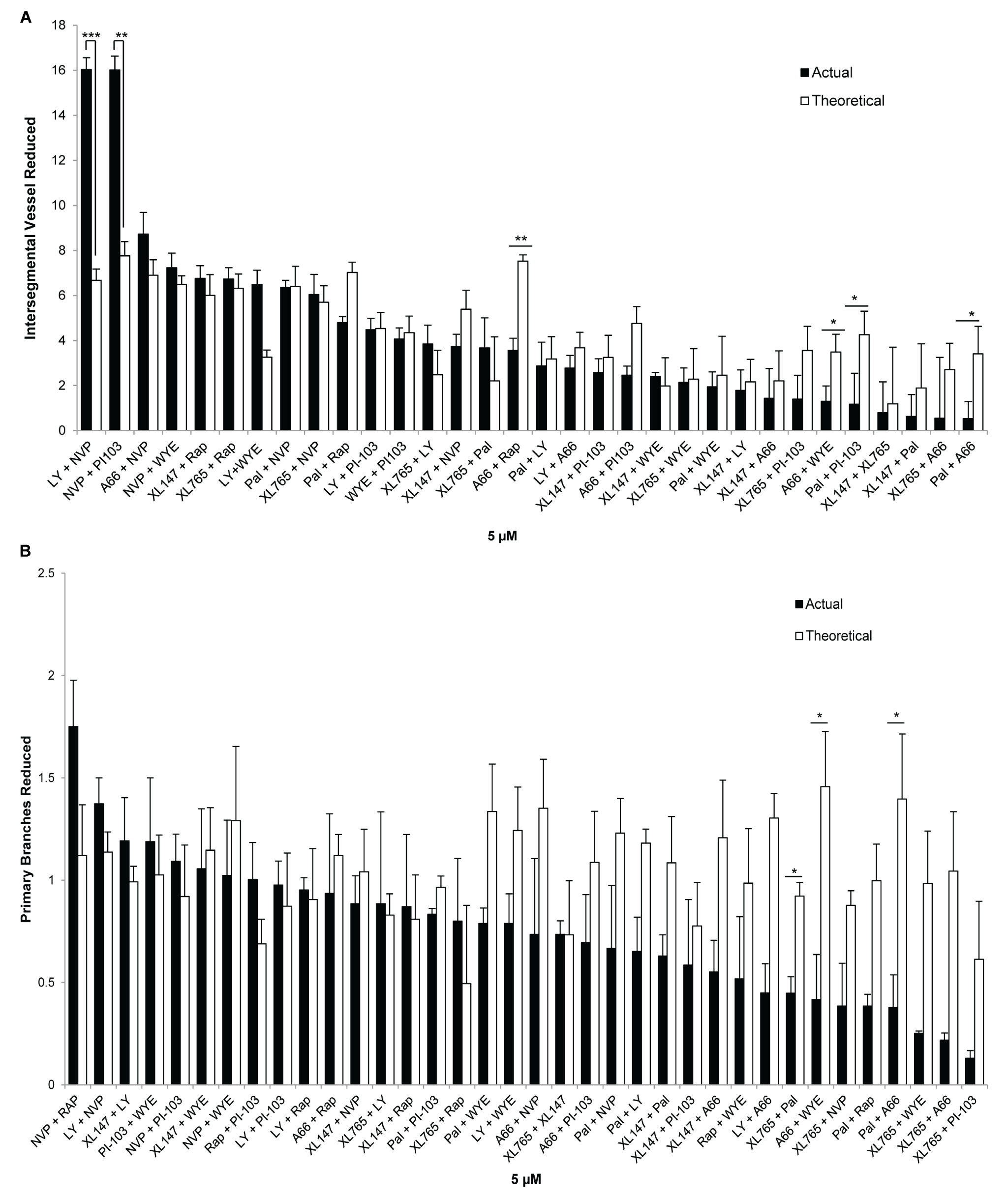

Supplement: Figure S1 — Combination of PI3K/Akt/mTOR inhibitors exhibit additive or greater than additive anti-angiogenic activity in vivo. Multiple PI3K pathway inhibitors were screened for effects on developmental angiogenesis in vivo. Tg(fli1:EGFP) embryos were treated with drugs alone or in combination from 6–48 hpf (ISV) or from 2–5 dpf (HV). Bar graphs show the ranking efficacy of “experimental observed” versus “theoretically calculated” responses in the ISV (A) and HV (B) assay. Experimental data (black bar) represent response from 5 µM of an inhibitor combination tested together, while theoretical data (white bar) represents the sum of the responses of the corresponding drug pair tested singly. Those combinations exhibiting anti-angiogenic responses significantly greater than the calculated additive responses are marked by asterisks. Four combinations, 5 µM A66 + Rapamycin, 5 µM A66 + WYE-125132, 5 µM Palomid 529 + PI103 or 5 µM Palomid 529 + A66 exhibit significantly reduced ISV responses compared to their calculated additive response, indicating drug antagonism. Three combinations, 5 µM LY294002 + A66, 5 µM Palomid 529 + A66 or 5 µM A66 + WYE-125132 appear to have antagonist effects in the HV assay. Data are means ± s.e.m (n = 24–30). *P<0.05, **P<0.01 & ***P<0.001. (TIF) [file pone.0105280.s001.tif]

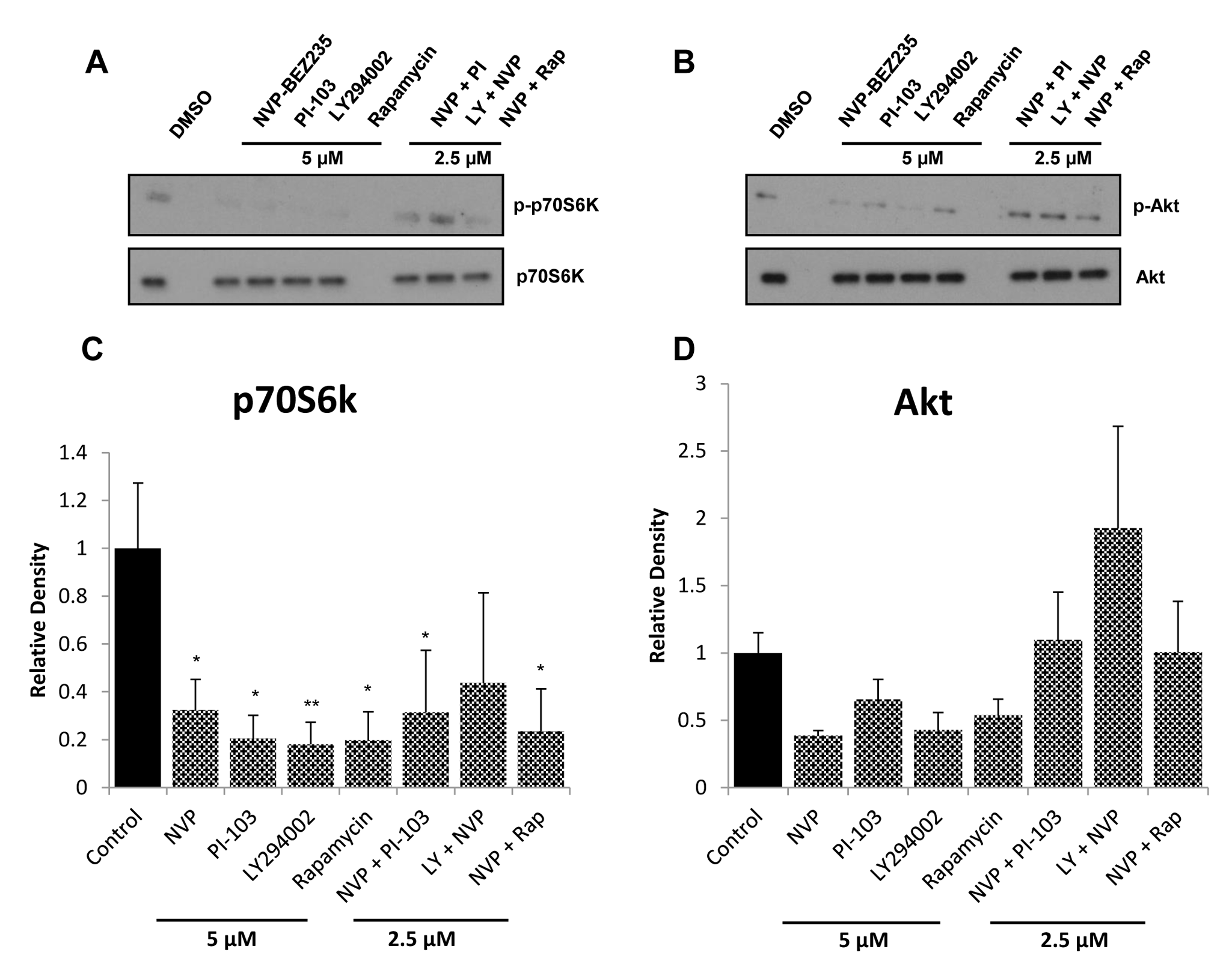

Supplement: Figure S2 — PI3K/Akt/mTOR inhibitors attenuate p70S6k and Akt activity in zebrafish larvae. Representative Western blot (A–B) and densitometric analysis (C–D) of extracts from whole larvae treated from 2–5 dpf with 5 µM NVP-BEZ235, 5 µM PI-103, 5 µM LY294002, 5 µM Rapamycin, 2.5 µM NVP-BEZ235 + PI-103, 2.5 µM LY294002 + NVP-BEZ235 and 2.5 µM NVP-BEZ235 + Rapamycin or DMSO. The vast majority of individual and combination drugs significantly reduced the levels of the downstream target p-p70S6k (Thr389) with only modest or no reductions in the levels of p-Akt (Thr308) which is further upstream. Bar graphs show mean band density normalized relative to p-p70S6k/p70S6k ratio or p-Akt/Akt ratio. Data are means ± s.e.m of 4 independent experiments (n = 60–80). *P<0.05 & **P<0.01 relative to vehicle control. (TIF) [file pone.0105280.s002.tif]

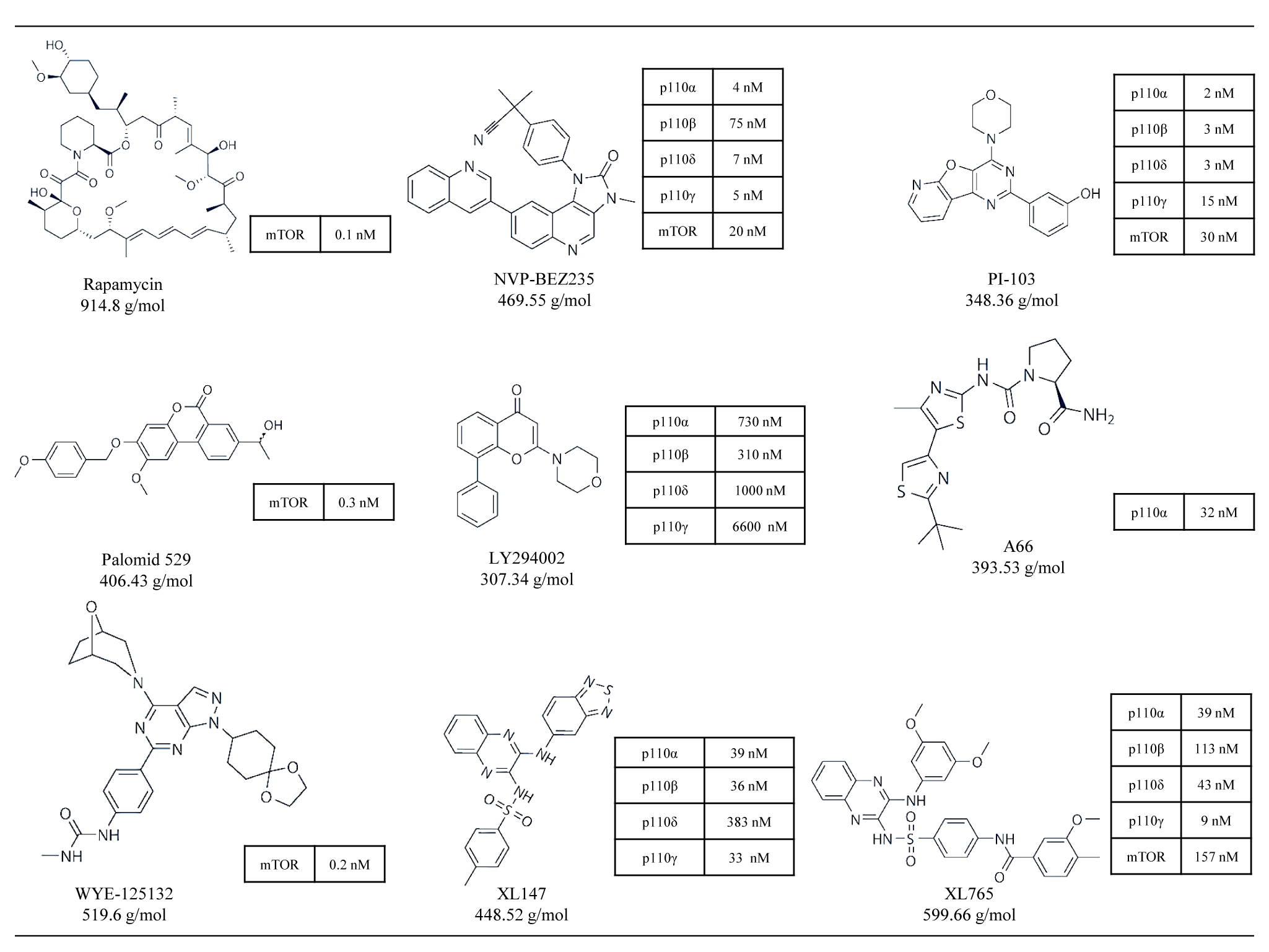

Supplement: Figure S3 — Chemical structures, molecular weight and IC50 values of screened PI3K/AKT/mTOR pathway inhibitors. (TIF) [file pone.0105280.s003.tif]
